# TiO_2_/Karaya Composite for Photoinactivation of Bacteria

**DOI:** 10.3390/ma15134559

**Published:** 2022-06-28

**Authors:** Anderson C. B. Lopes, Francisca P. Araújo, Alan I. S. Morais, Idglan S. de Lima, Luzia M. Castro Honório, Luciano C. Almeida, Ramón Peña Garcia, Edson C. Silva-Filho, Marcelo B. Furtini, Josy A. Osajima

**Affiliations:** 1 Interdisciplinary Laboratory Advanced Materials (LIMAv), Federal University of Piauí, Teresina 64049-550, PI, Brazil; andersoncastellobranco@gmail.com (A.C.B.L.); araujofp15@gmail.com (F.P.A.); alanicaro@gmail.com (A.I.S.M.); i.dglan@hotmail.com (I.S.d.L.); edsonfilho@ufpi.edu.br (E.C.S.-F.); marcelofurtini@ufpi.edu.br (M.B.F.); 2 Department of Chemistry and Physics, Center for Agrarian Sciences, Federal University of Paraíba, Areia 58397-000, PB, Brazil; luzia_quimica@yahoo.com.br; 3 Chemical Engineering Department, Federal University of Pernambuco, Recife 50670-901, PE, Brazil; luciano.calmeida@ufpe.br; 4 Academic Unit of Cabo de Santo Agostinho, Federal University of Pernambuco, Cabo de Santo Agostinho 50670-901, PE, Brazil; rraudelp@gmail.com

**Keywords:** photocatalyst, Karaya gum, reactive oxygen species, *Escherichia coli*, *Staphylococcus aureus*

## Abstract

TiO_2_/Karaya composite was synthesized by the sol-gel method for the photoinactivation of pathogens. This is the first time that we have reported this composite for an antimicrobial approach. The structure, morphology, and optical properties were characterized by X-ray diffraction (XRD), scanning electron microscopy (SEM), energy dispersive X-rays (EDS), Fourier transform infrared spectroscopy (FTIR), and diffuse reflectance, and the surface area was characterized by the BET method. The XRD and EDS results showed that the TiO_2_/Karaya composite was successfully stabilized by the crystal structure and pore diameter distribution, indicating a composite of mesoporous nature. Furthermore, antibacterial experiments showed that the TiO_2_/Karaya composite under light was able to photoinactivate bacteria. Therefore, the composite is a promising candidate for inhibiting the growth of bacteria.

## 1. Introduction

With advances in technology and the growing population without adequate planning, the limitless release of residual effluents is directly impacting the environment and represents an eminent concern in environmental management policy [1,2]. In addition, high concentrations of micropollutants, organic/synthetic chemicals, pesticides, heavy metals, and other emerging contaminants are significant concerns for human health and environmental safety [3,4].

Among the emerging pollutants, antibiotics are natural or synthesized substances that act to inhibit bacterial growth. They are formulated to destroy the growth of bacteria and treat infections by their antimicrobial potential [3,5]. However, they present different mechanisms of action and chemical structures that make it challenging to remove them from aquatic matrices, making them persistent, according to the World Health Organization (WHO) [3,6]. Although they have inhibitory functions, antimicrobial contamination favors the emergence of bacteria that are highly resistant to antibiotics [7]. Examples of bacteria include *Escherichia coli* and *Staphylococcus aureus*, which differ in terms of their cell walls and cause different infections. The investigation of photocatalysts to combat pathogenic microorganisms through photocatalytic processes has been conducted. This process guarantees a photochemical mechanism that can cause changes in the cell membrane that cause the death of bacteria [8,9,10,11].

Inorganic oxides with antibacterial performance have been essential in the current scenario. The presence of irradiation (visible light, fluorescent lamps, and LED) has the ability to inactivate several pathogenic microorganisms due to reactive oxygen species (ROS). They can promote oxidative stress and, consequently, disrupt the bacterial growth structure through electrostatic interactions, inactivating cellular functions [12,13,14]. Furthermore, the production of ROS (O_2_^•−^**,** HO_2,_^•^OH, and H_2_O_2_) in the reaction medium occurs through photochemical mechanisms based on electron transfer to form oxygen radicals and/or by direct energy transfer among the photosensitizing materials that produce singlet oxygen (^1^O_2_), which is considered toxic to bacterial cells [15,16]. According to Yao et al. [17], ROS generation contributes to optimizing and improving antibacterial performance in chemically modified TiO_2_ nanoparticles, reinforcing its essential role in cell survival, cell death, cell differentiation, and inflammations. 

Due to its effectiveness and attractive properties, such as chemical stability, selectivity, biocompatibility, and non-toxicity, titanium dioxide (TiO_2_) is highly functional in the degradation of organic pollutants [18], as well as in the treatment of various bacterial pathogens [19,20,21]. TiO_2_ can be chemically modified through surface strategies (immobilization, deposition, doping, and stabilization) that optimize its deficiencies and facilitate its antibacterial performance [14,22,23]. Yao et al. [17] showed that the heterojunction between nanoparticles (SnSO_4_-modified TiO_2_) significantly promoted the inhibition of *E. coli* and *S. aureus*. In addition, the enhanced photoinactivation of sulfur-doped TiO_2_ nanoparticles under visible light to combat the spread of *Vibrio cholerae* was reported by Tariq et al. [24]. Studies of antibacterial activity showed that the doping of other synthesized membranes with silver nanoparticles significantly increased the inhibition of *E. coli* bacteria.

Stabilization using natural gums is considered an effective strategy to improve the mobility of charge carriers and light responses [25]. Furthermore, gums are examples of green eco-materials with high availability and low cost [26]. Therefore, they are more efficient in terms of structural changes, as reported by Araujo et al. [25,27,28], who combined oxides to degrade synthetic dyes under visible irradiation. This work aimed to synthesize a TiO_2_/Karaya composite, called GKT, for the photoinactivation of bacteria. This is the first time that we have reported this composite, TiO_2_/Karaya, for an antimicrobial approach. This study used Gram-positive *Staphylococcus aureus* ATCC 25923 (SA) and Gram-negative *Escherichia coli* bacteria ATCC 25922 (EC). The composite was synthesized by the sol-gel method and characterized by XRD, SEM, Diffuse Reflectance, and FTIR. Finally, the mechanistic responses of inactivation and growth against infection-causing pathogens were based on the action of reactive oxygen species (ROS). 

## 2. Materials and Methods

### 2.1. Materials 

The reagents used to synthesize the photocatalysts were Karaya Gum (KG)—Lot SLBP5629V (Aldrich, St. Louis, MO, USA), ethyl alcohol 99.8% (Aldrich), titanium isopropoxide 97% (Aldrich), methylene blue 97% (Dinâmica, Indaiatuba, SP, Brazil), and ultrapure water. For antibacterial tests, the reagents used were Brain Heart Infusion and Mueller Hinton media (HIMEDIA, Mumbai, MA, India) and Mueller Hinton with sodium chloride (IMPEX, Kolkata, WB, India).

### 2.2. Synthesis of TiO_2_/Karaya Composite (GKT)

GKT was synthesized by the sol-gel method as previously reported [25,27,29] with some modifications. More detail about this modification is provided in the supplementary materials. A mixture of 2% of Karaya Gum (KG) against the volume of the titanium with 100.0 mL of ethyl alcohol was kept under stirring for 30 min. Then, 6.0 mL of titanium isopropoxide was slowly added to the gum solution under magnetic stirring. After 30 min, 6.0 mL of ultrapure water was slowly added and allowed to stir for a further 30 min. Afterward, the solution was left to rest for 24 h and then dried inside an incubator at 75 °C. Finally, the GKT was calcined at 400 °C.

### 2.3. Physico-Chemical Characterization

The crystal structure was investigated by X-ray diffraction in a scanning range of 2θ = 3° to 75° using an X-ray diffractometer Shimadzu, model Labx-XDR 6000, with Cu-Kα radiation (λ = 1.5406 Å in the Bragg–Betano configuration. Scanning Electron Microscopy (SEM) using a scanning electron microscope with an electron source by field emission FEG (Field Emission Gun), Quanta FEI 250. Spectroscopy in the Fourier Transform Infrared Region (FTIR) was performed on a Perkin Elmer SPECTRUM 400 (FTIR/FT-NIR) spectrometer with a sweep from 4000 to 400 cm^−1^. The textural properties of the solids were investigated based on the nitrogen adsorption–desorption isotherms using Quantachrome (Autosorb-iQ Instruments) results. The surface area, pore volume, and diameter were calculated using the Brunauer–Emmett–Teller (BET) method based on N_2_ adsorption–desorption. The material’s bandgap (Eg) was determined using a Shimadzu spectrophotometer Model UV-3600 with a diffuse reflectance accessory monitoring the region of 200 to 800 nm and was calculated through a series of mathematical transformations proposed in the Kubelka–Munk method.

### 2.4. Photoinactivation of Bacteria

Standard strains of Gram-positive *Staphylococcus aureus* ATCC 25923 (SA) and Gram-negative *Escherichia coli* ATCC 25922 (EC) were used to perform the antibacterial activity test. Both were provided by the Microbiology Research Laboratory of the Federal University of Piauí. The bacterial cultures were obtained by transferring the inoculum from the bacterial growth stock to Brain Heart Infusion (BHI) medium and incubated at 37 °C for 24 h. After the incubation period, the inoculum suspension was standardized in saline solution at 1.5 × 10^8^ colony-forming units per mL (CFU/mL). After preparing the standard inoculum, 1 mL of this suspension was added to Eppendorf containing 10 mg of the TiO_2_/Karaya composite [30]. Then, the Eppendorf containing the suspension with the material was separated into two groups, under darkness and under light. Irradiation occurred for 2 h under a UV-Vis light source–neutral white LED system (4000–4500 K) LK1230_M001. The irradiation process took place both for the solution containing the bacterial strains and TiO_2_/Karaya, and for the solution containing only the bacterial strains. The control and the samples were left in the dark to compare process efficiency. Finally, 100 μL of the solutions was transferred to Petri dishes containing Mueller Hinton agar medium and seeded with a loop of Drigalski. Bacterial viability was determined by a direct contact test in a solid medium [31]. Subsequently, the antimicrobial activity followed the same methodology of the experiments carried out under darkness with incubation at 37 °C for 24 h [11].

**Figure 1:**
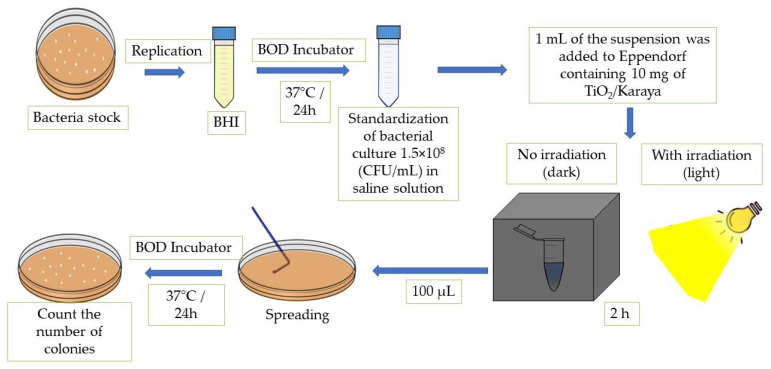
Scheme of the methodology for the photoinactivation tests.

The inactivation of bacteria by each test solution was calculated by Equation (1).

(1) $$Ƞ=\frac{\left(N_{1}-N_{2}\right)}{N_{1}}$$

where ƞ is defined as the inactivation of bacteria, N_1_ is the arithmetic average of the number of CFU of the control plates, and N_2_ is the arithmetic average of the number of CFU of each tested solution. The method described was carried out individually for each proposed strain. Figure 1 displays the scheme of the methodology for the photoinactivation tests.

## 3. Results and Discussion

### 3.1. Physico-Chemical Characterization

**Figure 2:**
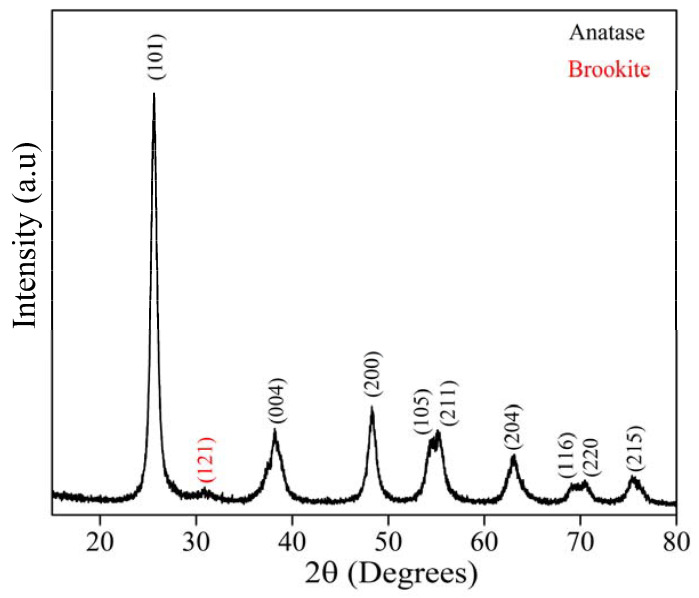
XRD pattern of TiO_2_ synthesized in the presence of Karaya Gum calcinated at 400 °C.

Figure 2 shows the XRD pattern for the GKT samples. The diffraction peaks at 25°, 38°, 48°, 54°, 55°, 63°, 69°, and 70°, indexed to the (101), (004), (200), (105), (211), (204), (116), and (220) planes, respectively, belonged to the anatase phase of TiO_2_ and were identified by the Crystallographic Open Database (reference code: 96-101-0943), while the emerging peak at 31° was associated with the brookite (121) phase (reference code: 96-900-4138). For the compound synthesized from natural reagents with a calcination temperature of 400°, the XRD pattern suggested the formation of a crystalline structure due to the narrow and well-defined peaks’ presence [32,33,34,35,36]. Using the Williamson and Hall equation, the average crystallite size (*D*) and lattice strain (*ε*) were calculated for the majority anatase phase. The *D* and *ε* values were 10 nm and 1.65 %, respectively, being close to the reported in the literature for TiO_2_ synthesized by the sol-gel method under other conditions [37].

**Figure 3:**
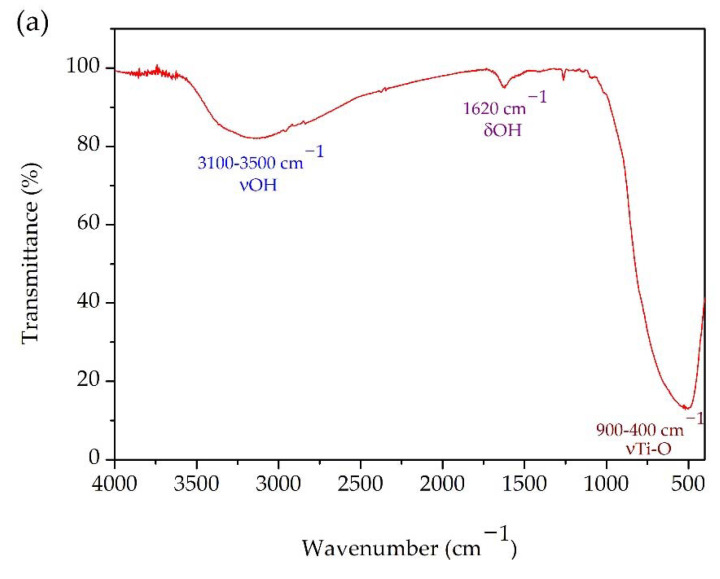

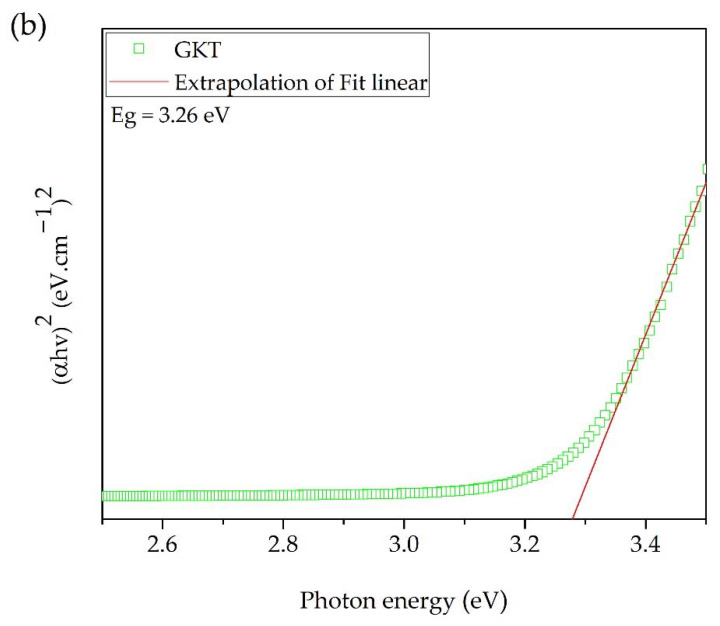
(**a**) FTIR spectrum and (**b**) bandgap value (Eg) of GKT determined according to the Kubelka–Munk method.

The functional groups present in the GKT were investigated by FTIR, as shown in Figure 3a. The band between 3100 and 3500 cm^−1^ was attributed to the bond vibration of the stretching of the hydroxyl groups [36,38]. The deformation vibrations of the hydroxyl group are present around 1620 cm^−1^ [39,40,41]. Studies have reported that the bands between 900 and 400 cm^−1^ are the characteristic bands of TiO_2_ [40,42,43]. The UV–Vis reflectance value was used to determine the optical band gap energy (E_g_) for the TiO_2_/Karaya composite. E_g_ was determined by extrapolating the linear part of the plot of (αhν)^2^ versus hν, as shown in Figure 3b. The reflectance spectrum is shown in the supplementary material (Figure S1).

**Figure 4:**
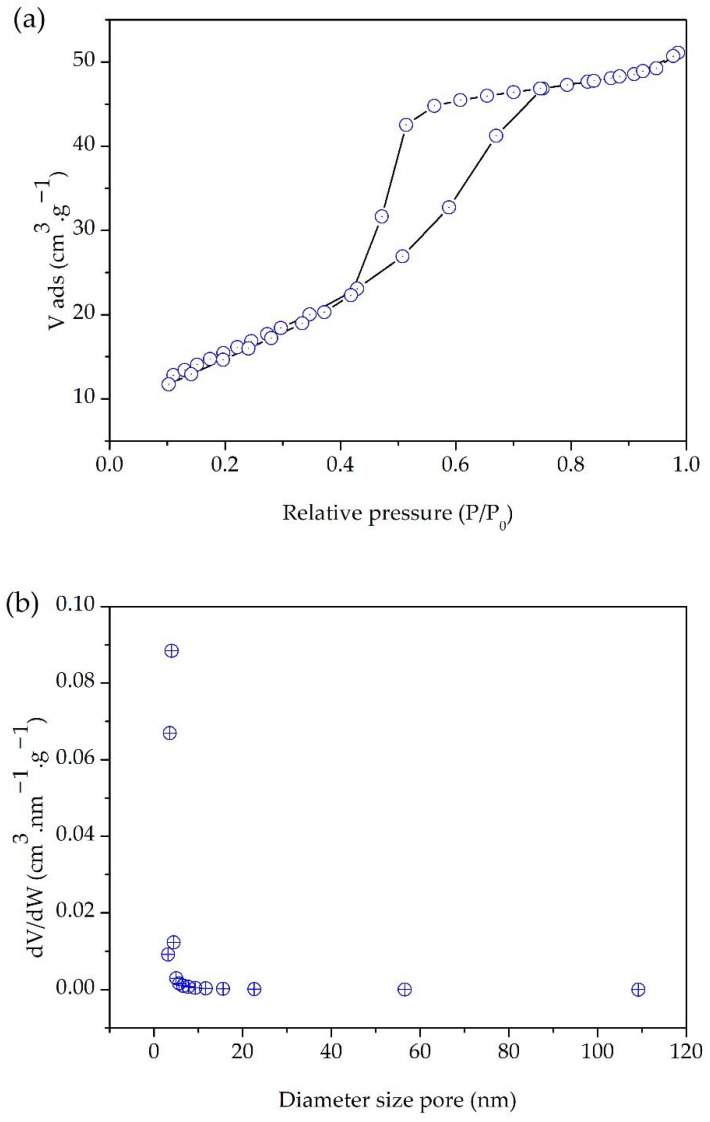
(**a**) N_2_ adsorption–desorption isotherm curve; (**b**) pore size distribution of GKT.

The nitrogen adoption–desorption isotherms of the GKT are shown in Figure 4a,b. The isotherm was similar to the IUPAC type-IV classification, which indicated the existence of a mesoporous structure [44,45,46]. Table 1 shows the surface area, pore volume, and pore size of the GKT. The pore diameter had an approximate value of 5 nm. Figure 4b displays the distribution of pore diameters between 2–50 nm, confirming the classification of mesoporous materials. GKT had a surface area of 38 g m^2^ g^−1^. This information is shown in Table 1.

**Figure 5:**
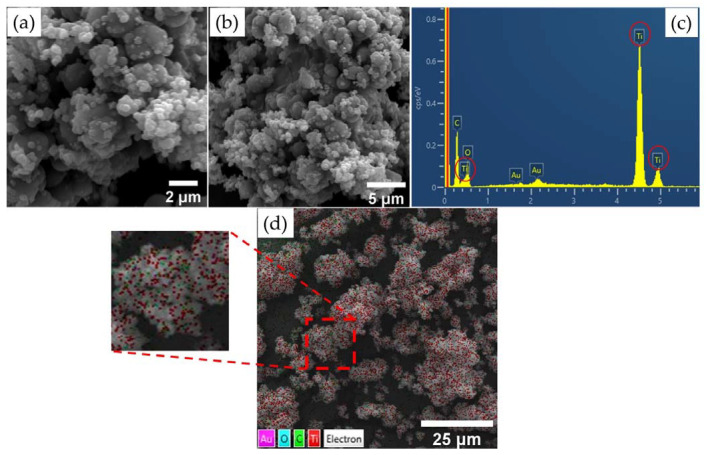
GKT: (**a**) SEM and semi-quantitative analysis at a scale of 5 µm, (**b**) SEM and semi-quantitative analysis at a scale of 2 µm, (**c**) EDS mapping, (**d**) and elemental mapping.

The morphological analysis results are shown in Figure 5a,b,d. Both images demonstrate the spherical shape of the nanoparticles, and the average diameter of the composite was 0.128 μm ± 0.05. In addition, it was observed that the sample exhibited small agglomerates. The EDS analysis results are shown in Figure 5c. The peaks referring to TiO_2_ demonstrate that the composite was synthesized with success and indicate the purity of the material. The peak indicating C refers to the double-sided tape used for the deposition and fixation of the material. Figure 5d shows the Ti and O particles present in the GKT, with Ti represented by the color red and O represented by cyan.

### 3.2. Photoinactivation of Bacteria

**Figure 6:**
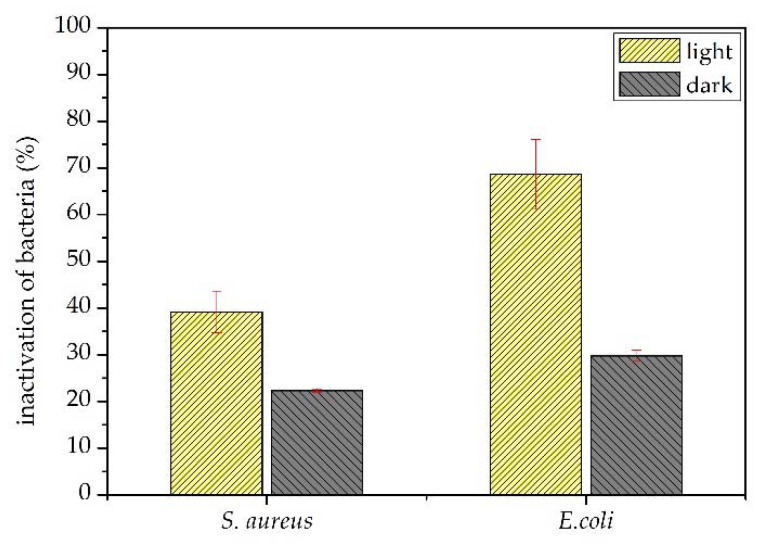
Inactivation of TiO_2_/Karaya against Gram-positive and Gram-negative bacteria under darkness and under light.

Figure 6 shows the antibacterial activity of the TiO_2_/Karaya composite against Gram-positive and Gram-negative bacteria. GKT presented an antibacterial inhibitory effect of around 20% for *S. aureus* strains and 29% for *E. coli* strains. The antibacterial activity results demonstrate the inhibitory effect under light. The inactivation of *S aureus* strain bacteria could be explained by the oxidative action of the peptidoglycan layer and the *E. coli* strain by lipid peroxidation. Both produced reactive oxygen species when GKT was irradiated under light [47]. The TiO_2_/Karaya composite exhibited photoinactivation of around 40% against *S. aureus* and 70% against *E. coli*. The production of radicals is based on the photocatalytic process. 

Photocatalysis occurs when a semiconductor is irradiated with enough energy to promote the electron (e^−^) from the valence band (VB) to the conduction band (CB). This process promotes electrons (e^−^) to the conduction band and generates a hole (h^+^) in the valence band (Equation (2)). The hole produces very positive potentials that form hydroxyl radicals from the reaction with water (Equation (3)). The e^−^ reacts with the O_2_ adsorbed in water to form O_2_^−^ radicals (Equation (4)). H^+^, e^−^, and OH are the main ROS responsible for bacterial photoinactivation and the degradation of organic pollutants (Equation (5)) [3,48,49]. The degradation of methylene blue proved the formation of ROS, which were responsible for the photoinactivation of bacteria. The model molecule’s kinetic photodegradation is displayed in the supplementary material (Figure S2).

(2) $$\mathrm{GKT}+h\nu \;\to \mathrm{GKT}+e^{-}+h^{+}$$

(3) $$H_{2}O+h^{+}\to \;\cdot \mathrm{OH}+{\;H}^{+}$$

(4) $$O_{2}+e^{-}\to {\;O}_{2}^{-}\;$$

(5) $$\mathrm{cell}\;\mathrm{membrane}+h^{+}+\cdot \;\mathrm{OH}+{\;O}_{2}^{-}\to \mathrm{photoinactivation}\;\mathrm{bacterial}$$

ROS are responsible for bacterial inactivation by oxidizing membranes, proteins, lipids, and genetic material [50]. However, Gram-negative and Gram-positive strains have structural differences in their cell walls. Gram-positive strains have multiple layers of peptidoglycan, resulting in a thicker layer populated with lipoic and teichoic acids. On the other hand, Gram-negative bacteria have a polysaccharide membrane above the peptidoglycan layer [47,51]. Upon semiconductor activation, the ROS oxidize the peptidoglycan layer and the polysaccharide layer, facilitating the reduction in cell viability in the system [52,53]. Cell membrane destruction can also occur through the interaction between microbes and particles from the deposition of bacteria on the semiconductor agglomerate [30].

The TiO_2_/Karaya composite exhibited the anatase phase of the titanium matrix, as shown in Figure 2. TiO_2_ has three types of crystal structure: rutile, anatase, and brookite. Among the polymorphic types of TiO_2_, anatase is the phase that has the best photocatalytic performance [36,54]. The calcination temperature during the synthesis process directly influences the material’s crystal structure. The anatase phase is formed at temperatures ranging between 400 and 700 °C. The FTIR presented in Figure 3 did not present any functional groups different from those of TiO_2_, indicating that the gum was eliminated in the calcination process. Figure 4a,b demonstrates the formation of a mesoporous material. Depending on the average diameter, pores are classified as micropores (<2 nm), mesopores (2–50 nm), and macropores (>50 nm) [55].

Figure 6 shows the results of the bacterial photoinactivation assay for *S. Aureus* and *E. coli* in the presence of light and under dark conditions. The bacteria irradiated by light did not undergo bacterial inactivation, demonstrating that the lamp used did not influence the process of destroying the cell membrane of the bacteria. The inhibitory effect was observed when there was a photocatalyst. Under dark conditions, the bacterial inactivation was around 20% for *S. aureus* strains and 29% for *E. coli* strains. The direct interaction between bacteria and GKT showed a better inhibitory effect on *S. Aureus* bacteria. The thicker peptidoglycan layer offered more excellent resistance to the TiO_2_/Karaya composite than the thin layer of peptidoglycan in *E.coli* around the lipopolysaccharide layer (LPS) [52].

Several TiO_2_-based composites with antibacterial properties under dark conditions have been reported in the literature, as shown in Table 2. The morphology of the material is an essential factor for evaluating its antibacterial potential. TiO_2_-based nanoparticles (NPs) were previously investigated for the influence of size and their antibacterial properties [30]. Smaller nanoparticles have better antibacterial activity [30,56]. In this study, the average diameter of the composite was 0.128 μm ± 0.05, as illustrated in Figure 4. This indicates that the smaller aggregates influenced the antibacterial approach of the material. Anandgaonker et al. synthesized TIO_2_ nanoparticles by an electrochemical method varying the current density from 10 mA/cm^2^ to 14 mA/cm^2^, resulting in particles with average crystalline sizes of 25 nm and 20 nm, respectively. The samples synthesized with a current of 14 mA/cm^2^ showed a better antibacterial activity, indicating that smaller crystallite sizes achieve better results [57]. Manjunath et al. synthesized TiO_2_ nanoparticles with an average crystallite size and average nanoparticle size of 40 nm and 35 nm, respectively. The nanoparticles showed antibacterial activity against Gram-negative (*Klebsiella aerogenes*, *Pseudomonas desmolyticum*, and *E. Coli*) and Gram-positive strains (*S. aureus*) [42]. Li et al. synthesized TiO_2_/chitosan composites that exhibited growth reduction of 49.4% and 74.6% in an antibacterial assay with *Xanthomonas oryzae pv. Oryzae* strain with and without extracellular polysaccharides, respectively [58].

**Figure 7:**
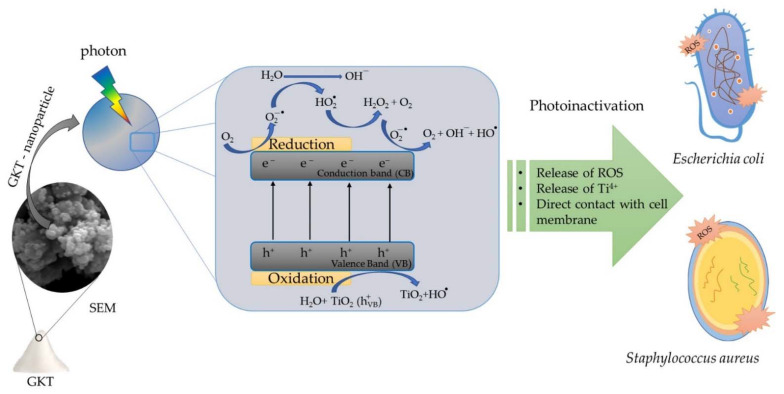
Photoinactivation of GKT against Gram-positive and Gram-negative bacteria.

Therefore, the TiO_2_/Karaya composite (GKT) in the presence of light showed promising bacterial photoinactivation results. The results indicate that the TiO_2_ synthesized with Karaya gum presented antibacterial activity of around 40% for *S. aures* and 70% for *E. coli*. ROS oxidized *E.coli* LPS, quickly destroying the thin peptidoglycan layer. The destruction of the cell wall allowed bacterial inactivation, providing the intracellular oxidation of the bacteria. However, intracellular oxidation occurs by DNA denaturation due to an increased catalyst particle size. The reduction of cell viability will occur due to the leaching of K^+^ ions and the breakage of protons, directly influencing the cell osmotic activity and the synthesis of adenosine triphosphate (ATP), respectively. Figure 7 summarizes the processes of the photoinactivation of bacteria using GKT as a photocatalyst.

## 4. Conclusions

TiO_2_/Karaya composite was successfully synthesized by the sol-gel method. The XRD showed the formation of a highly crystalline material exhibiting the anatase phase of TiO_2_. The characteristic bands from the FTIR spectra indicated the presence of TiO_2_. SEM showed that the composite was nanoparticles and had agglomerates. The isotherms of nitrogen adsorption and desorption and the average pore size distribution result indicated mesoporous structures. The TiO_2_/Karaya composite (GKT) presented antibacterial activity under dark and light conditions. The better inactivation of bacteria occurred under light, and the production of ROS can explain why this behavior was able to photo-inactivate bacteria. Therefore, the TiO_2_/Karaya composite (GKT) is a promising material for antibacterial applications. 

## Figures and Tables

**Table 1:** BET parameters of TiO_2_/Karaya composite.

Composite	Surface Area (m^2^ g^−1^)	Average Pore Diameter (nm)	Pore Volume (cm^3^ g^−1^)
GKT	38.5	5.09	0.073

**Table 2:** TiO_2_-based composite, synthesis method, and bacterial strain studied.

Samples	Method	Bacteria	References
TiO_2_ nanoparticles	Sol-gel	*E. coli*/ *S. aureus*	[30]
TiO_2_ nanoparticles	Electrochemical	*E. coli*/ *S. aureus*	[57]
TiO_2_ nanoparticles	Hydrothermal	*Klebsiella aerogenes*/*E. Coli*/*Pseudomonas desmolyticum*/ *S. aureus*	[42]
TiO_2_/ chitosan	Sol-gel	*Xanthomonas oryzae pv. oryzae*	[58]

## Data Availability

Not applicable.

## Supplementary Materials

The following supporting information can be downloaded at: https://www.mdpi.com/article/10.3390/ma15134559/s1, Figure S1: Reflectance spectrum of TiO_2_/Karaya composite (GKT), Figure S2: Photocatalytic degradation of methylene blue.
