# Supplementary material for: TiO2/Karaya Composite for Photoinactivation of Bacteria
Source: Materials (Basel). 2022 Jun 28;15(13):4559. doi: 10.3390/ma15134559 (PMC9267801; doi:10.3390/ma15134559)
Supplement: Supplementary file 1 [file materials-15-04559-s001.zip › materials-1776908-supplementary.pdf]

TiO_2/Karaya composite for photoinactivation of bacteria

Anderson Lopes [1], Francisca P. Araújo [1], Alan I. S. Morais [1], Idglan S. de Lima [1], Luzia M. Castro Honorio[2], Luciano C. Almeida[3], Ramón Peña Garcia[4], Edson C. Silva-Filho[1], Marcelo B. Furtini[1] and Josy A. Osajima[1]

[1]Federal University of Piauí, Interdisciplinary Laboratory Advanced Materials (LIMAv), Teresina, PI, Brazil; andersoncastellobranco@gmail.com; araujofp15@gmail.com; alanicaro@gmail.com; i.dglan@hotmail.com; edsonfilho@ufpi.edu.br; marcelofurtini@ufpi.edu.br.

[2] Department of Chemistry and Physics - Center for Agrarian Sciences, UFPB, Areia, PB, Brazil; luzia_quimica@yahoo.com.br.

[3] Federal University of Pernambuco, Chemical Engineering Department, Recife, PE, Brazil; luciano.calmeida@ufpe.br

[4] Federal University of Pernambuco, Unidade Acadêmica do Cabo de Santo Agostinho, Cabo de Santo Agostinho-PE, Brazil, ;rraudelp@gmail.com

* Correspondence: josyosajima@ufpi.edu.br

Supplementary Material

More detail of Sysnthesis of TiO_2/Karaya composite (GKT)

TiO_2/Karaya gum composite was obtained by the sol-gel method. Initially, Karaya gum (2% w/v, relative to the volume of the titanium precursor) was added to a 100.0 mL ethyl alcohol and kept under stirring for almost 30 minutes. After, 6.0 mL of titanium isopropoxide was slowly added to the solution gum under vigorously magnetic stirring. After 30 minutes, 6.0 mL of ultrapure water was slowly added to the reaction and stirred for more than 30 minutes. The resulting material was aged for 24 hours, and the solid was resuspended, then centrifuged at 5000 rpm, dried under a stove at 70 °C, and the TiO_2 composite was calcined at 200 °C [25]. In our work, the only difference was that GKT was calcined at 400 °C.

Reflectance spectrum of TiO_2/Karaya composite (GKT)

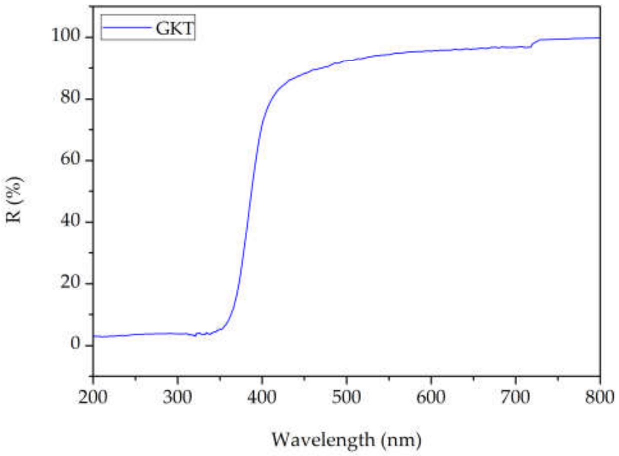

Figure S1. Reflectance spectrum of TiO_2/Karaya composite (GKT).

Photocatalytic performance

The dye methylene blue (MB) was used to evaluate the photocatalytic capacity of the materials. The concentration used was $1.5x10^{-5}$ $molL^{-1}$. The photodegradation was performed by a radiation box containing a borosilicate reactor at 25 °C. The light used as a light source was LED system-neutral white (4000-4500K) LK1230_M001. The samples were removed at predetermined times (0, 10, 30, 60, 120, and 150 min). The amount of photocatalyst was 1.0 g L^{-1}. Before the irradiation, the adsorption-desorption equilibrium in the dark was 60 min. Changes in MB concentration were monitored using an Agilent Technologies Cary 60 UV-Vis spectrophotometer by the absorption band at 663 nm, corresponding to the maximum absorption.

$$\% \text{ rate degradation } = \frac{(A\text{-}A_0)}{A} \tag{1}$$

Where A_0 and A correspond to the absorbances of the MB solution before and after irradiation, respectively, the kinetic study used the Langmuir-Hinshelwood model, and the rate constant, *k* was determined under pseudo-first-order. Figure S1 shows the kinetic of MB degradation using GKT as photocatalyst.

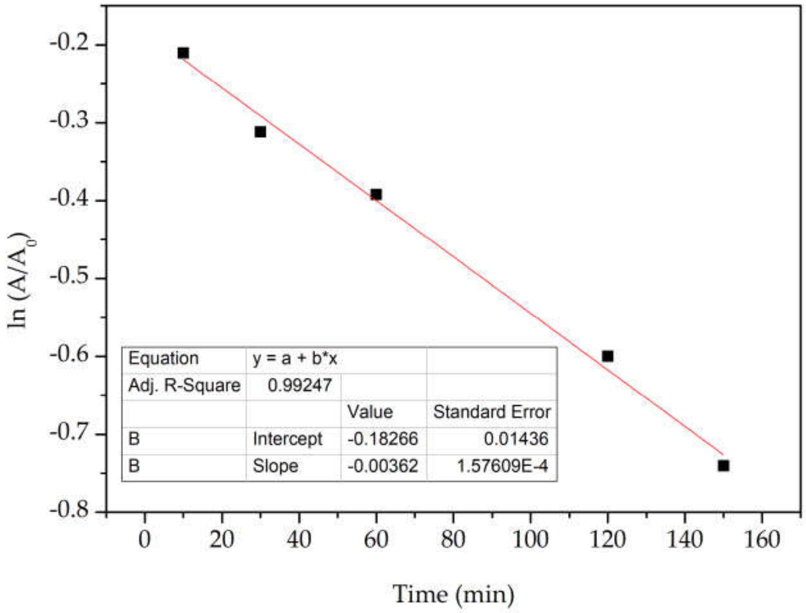

Figure S2. Photocatalytic degradation of methylene blue.
